# Effects of a Short-Term Lipopolysaccharides Challenge on Mouse Brain and Liver Peroxisomal Antioxidant and β-oxidative Functions: Protective Action of Argan Oil

**DOI:** 10.3390/ph15040465

**Published:** 2022-04-12

**Authors:** Soukaina Essadek, Habiba Bouchab, Riad El Kebbaj, Catherine Gondcaille, Soufiane El Kamouni, Stéphane Savary, Joseph Vamecq, Abdelkhalid Essamadi, Mustapha Cherkaoui-Malki, Boubker Nasser, Pierre Andreoletti

**Affiliations:** 1 Laboratoire Biochimie, Neurosciences, Ressources Naturelles et Environnement, Faculté des Sciences et Techniques, Université Hassan I, BP577, Settat 26000, Morocco; ess.soukaina@hotmail.fr (S.E.); habibabouchab78@gmail.com (H.B.); elkebbajriad@gmail.com (R.E.K.); eks.soufiane@gmail.com (S.E.K.); essamadi@uhp.ac.ma (A.E.); boubker_nasser@hotmail.com (B.N.); 2 Laboratoire Bio-PeroxIL EA7270, University Bourgogne Franche-Comté, 6 Bd Gabriel, 21000 Dijon, France; catherine.gondcaille@u-bourgogne.fr (C.G.); stsavary@u-bourgogne.fr (S.S.); 3 Laboratory of Health Sciences and Technologies, Higher Institute of Health Sciences, Hassan First University, Settat 26000, Morocco; 4 INSERM and HMNO, CBP, CHRU Lille, 59037 Lille, France; joseph.vamecq@inserm.fr; 5 RADEME EA 7364, Faculté de Médecine, Université de Lille 2, 59045 Lille, France

**Keywords:** argan oil, antioxidant, Acyl-CoA oxidase 1, brain, beta-oxidation, catalase, peroxisome, LPS, superoxide dismutase, glutathione peroxidase

## Abstract

During sepsis, the imbalance between oxidative insult and body antioxidant response causes the dysfunction of organs, including the brain and liver. Exposing mice to bacterial lipopolysaccharides (LPS) results in a similar pathophysiological outcome. The protection offered by argan oil was studied against LPS-induced oxidative stress, dysregulation of peroxisomal antioxidants, and β-oxidation activities in the brain and liver. In a short-term LPS treatment, lipid peroxidation (malonaldehyde assay) increased in the brain and liver with upregulations of proinflammatory *tumor necrosis factor* (*Tnf*)-*α* and anti-inflammatory *interleukin* (*Il*)-*10* genes, especially in the liver. Although exposure to olive oil (OO), colza oil (CO), and argan oil (AO) prevented LPS-induced lipid peroxidation in the brain and liver, only AO exposure protected against liver inflammation. Remarkably, only exposure to AO prevented LPS-dependent glutathione (GSH) dysregulation in the brain and liver. Furthermore, exposure to AO increased more efficiently than OO and CO in both organs, peroxisomal antioxidant capacity via induction of catalase (*Cat*) gene, protein and activity expression levels, and superoxide dismutase (*Sod1*) mRNA and activity levels. Interestingly, LPS decreased protein levels of the peroxisomal fatty acid-ATP binding cassette (ABC) transporters, ABCD1 and ABCD2, and increased acyl-CoA oxidase 1 (ACOX1) protein expression. Moreover, these LPS effects were attenuated for ABCD1 and ACOX1 in the brain of mice pretreated with AO. Our data collectively highlight the protective effects of AO against early oxidative stress caused by LPS in the brain and liver and their reliance on the preservation of peroxisomal functions, including antioxidant and β-oxidation activities, making AO a promising candidate for the prevention and management of sepsis.

## 1. Introduction

Sepsis is a life-threatening inflammatory disorder representing the immune response to infection, it is a leading cause of hospitalization, disability, and death worldwide [1,2]. Septic shock is linked to a broad spectrum of cerebral damage and dysfunction [1]. Following host exposure to endotoxins (lipopolysaccharides, LPS), an acute syndrome [3] may develop, which encompasses a cytokine response and a burst of reactive oxygen species (ROS) causing tissue injury [4]. In the host developing an acute sepsis syndrome, preservation of homeostasis depends on the body’s capacity to fine-tune and cope with the altered balance between inflammatory cytokine response, severe dysregulation of lipid metabolism, and yield of reactive oxygen species (ROS). Interestingly, peroxisome is a cell compartment containing at the same time oxidase enzymes involved in the generation of ROS and another set of enzymes able to metabolize H_2_O_2_ and other cell-damaging oxidant species [5]. One of the major peroxisomal functions is the β-oxidation of very-long-chain fatty acids (VLCFA). These VLCFA are transported through peroxisomal membrane by fatty acid ABC transporters ABCD1 or ABCD2. Then, these fatty acyl-CoAs are β-oxidized by the first enzyme ACOX1, which generates both enoyl-CoA and hydrogen peroxide (H_2_O_2_). Degradation of H_2_O_2_ is accomplished by the peroxisomal catalase [6]. In addition, ROS-scavenging peroxisomal enzymes include glutathione peroxidase (GPx), Cu-Zn superoxide dismutase (SOD), epoxide hydrolase, peroxiredoxin I, and peroxisomal membrane protein 20 [7]. This reveals that the balance between peroxisomal β-oxidative and antioxidative activities play a key role in cellular ROS homeostasis. Defects in peroxisome biogenesis or activity of peroxisomal enzymes may be associated with peroxisomal neurodegenerative diseases characterized by progressive demyelination [6,8]. The defect in peroxisomal β-oxidation system was reported in several leukodystrophies, leading to a deficiency in VLCFA degradation. Such deficiency was linked to a defect in ABCD1 peroxisomal VLCFA transport (X-linked adrenoleukodystrophy (X-ALD)) or metabolism (ACOX1 deficiency) [6,8]. In rat liver, LPS affects peroxisome functions through alterations of the peroxisomal membrane composition (both in fatty acid and phospholipid content) and the decreased peroxisomal proteins expression [9]. Previously, Khan et al. [9] have shown that LPS strongly repressed ACOX1 and the oxidation of VLCFAs in rat C6 glial cells. More recently, we have reported the reduced expression of genes involved in hepatic peroxisomal fatty acid oxidation in mice exposed to LPS [10].

Virgin edible argan oil (AO) is a vegetable oil extracted by cold-pressing roasted kernel of argan (*Argania spinosa (L.)* Skeels; Sapotaceae), an endemic tree to Morocco. However, culture of argan was principally disseminated in Israel, South Africa, and Tunisia. AO plays an important socio-economic role for the Amazigh population in the southwestern region of Morocco [11]. This oil comprises triacylglycerols (99%), containing balanced proportions of polyunsaturated fatty acids, principally oleic (44.8%) and linoleic acids (33.7%) [12]. Interestingly, the non-saponifiable fraction of AO, which constitutes 1% of total components, exhibits a high antioxidant activity [13,14]. In addition to phytosterols (Schottenol and Spinasterol) and phenols (ferulic, syringic, and vanillic acids), this fraction contains mainly tocopherols (α, β, γ and δ) present in a higher proportion than in olive oil [12,15].

Clinically, AO prevents cardiovascular disease [16], due to its unique composition of unsaturated fatty acids endowed with protective properties against LDL oxidation, stimulating properties towards reverse cholesterol transport, and increasing high-density lipoprotein (HDL) cholesterol level [13]. Epidemiological studies showed that regular consumption of edible olive oil could have significant protective effects against several types of cancer [17]. Since composition of argan and olive oils revealed common constituents with antioxidant and anti-inflammatory properties, an antiproliferative effect was attributed to argan oil [12]. Numerous studies have now highlighted the antioxidant effect of AO [13,16,18]. On the other hand, we previously reported in LPS-associated sepsis the beneficial effect of AO in preserving hepatic mitochondrial and peroxisomal activities, improving gluconeogenic gene expression, and recovering gene expressions of nuclear receptors PPARα and ERRα, and their coactivator PGC-1α [10]. Furthermore, we have shown that mice fed with standard chow supplemented with 6% (*w*/*w*) AO for 25 days protects against the dysregulation of antioxidant capacities and inflammatory status 16 h post-LPS injection [10].

AO’s liver antioxidant and anti-inflammatory effects were already explored [10,18,19,20]. To date, sepsis-associated brain peroxisome dysfunction has been reported [21,22], but a study of the potential protective effect of AO on the brain is still lacking. Here, we studied the short-term effect of LPS on the peroxisomal functions of the brain and liver in mice. The potential protective effect of AO against LPS was compared to other common edible oils, OO and CO. Thus, both OO and CO have different fatty acid compositions, regarding AO. In addition, OO is well-known as a Mediterranean diet component, while CO has become the most consumed oil in Europe [23]. The antioxidant capacity in the brain and liver was assessed by measuring antioxidant enzymes activities and reduced glutathione. The expression of genes and proteins related to the peroxisomal functions and those involved in oxidative stress and inflammation were also evaluated.

## 2. Results

The hepato-protective effect of AO after a 16 h LPS-induced sepsis (16 h) was described in previous reports [10,18]. Here, we attempted to evaluate the protective effect of AO on the brain and liver in a short-term 4 h post-LPS injection. The effects of argan oil and two additional standard edible oils, olive and colza, on several metabolic and peroxisomal antioxidant markers in both brain and liver were compared. Table 1 reported the chemical analysis of these oils, showing a unique fatty acid profile of AO with 32.2% of C18:2n−6 and 46.4% of C18:1n−9, while OO and CO reveal only 9.95% and 19% of C18:2n-6, and 76.35% and 63% of C18:1n-9, respectively. Two groups of mice received each for 28 days a standard chow supplemented or not with 6% (*w*/*w*) of one of the three compared oils (AO, OO, or CO). Four hours before euthanasia, mice from the first group received an injection of 100 µg LPS via tail vein, while the control group received only an injection of PBS.

### 2.1. Oxidative Stress and Inflammatory Biomarkers

**Figure 1:**
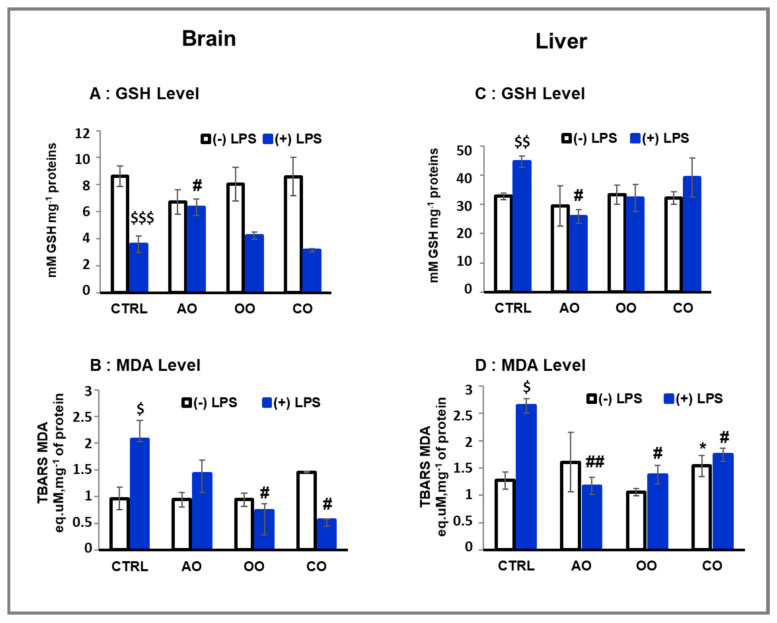
Effect of argan oil, olive oil, and colza oil treatments on GSH level (**A**,**C**) and lipid peroxidation (**B**,**D**) in brain and liver. Male C57BL/6 mice received for 28 days a standard diet (control: CTRL), a diet enriched with 6% (*w*/*w*) AO, OO, or CO, and an intravenous injection of LPS (100µg) four hours antemortem. All values are means ± SD (*n* = 3), Statistical significance of higher mean signal strength (* *p ≤* 0.05) compared to control, (## *p ≤* 0.01, # *p* ≤ 0.05) compared to LPS and ($$$ *p* ≤ 0.001, $$ *p ≤* 0.01, $ *p* ≤ 0.05) compared to the different treatments with or without LPS administration.

LPS-treated groups showed a significant decrease (41%; *p* < 0.001) in the brain GSH levels (Figure 1A). By contrast, the liver of LPS-treated mice revealed a significant increase (136%; *p* < 0.01) of GSH level compared to the control group (Figure 1C). In both brain and liver, treatment of mice with AO, OO, or CO did not affect the level of GSH. However, in both the brain and liver of LPS-treated mice, the pretreatment with argan oil maintained the GSH content at the level observed in the control mice (*p* < 0.05) (Figure 1A,C). In addition, although pretreatments with OO or CO helped to maintain liver GSH content near the control level, these oils failed to maintain control levels of GSH in the brains of LPS-treated mice (Figure 1A,C).

Lipid peroxidation was estimated by measuring the malonaldehyde (MDA) levels in the brains and livers from AO, OO, and CO and comparing them to the control group of mice. LPS administration led to a significant increase in MDA levels (215% and 207%. *p* < 0.05) in the mouse brain and liver, respectively (Figure 1B,D), compared to the control group. However, after 28 days of treatment, neither AO nor OO administration significantly affected MDA levels in both brain and liver tissues (Figure 1B,D). Only CO administration revealed a significant increase (120%. *p* < 0.05) in MDA levels in the liver compared to the mice control group (Figure 1B,D). Interestingly, LPS-dependent increase in MDA level was attenuated in the brain of animals pretreated with vegetal oil: AO-LPS group by 64% (*p* < 0.01), OO-LPS by 65% (*p* < 0.05), and by 73% (*p* < 0.05) in CO-LPS group. In conclusion, each of the three oils (AO, OO or CO) prevented the LPS-induced MDA level with a counteracting effect more important for CO and OO in the brain than in the liver of LPS-treated mice (Figure 1B,D). Both OO and CO have in common in their composition of β-sitosterol, by contrast to AO that does not contain this compound. Interestingly, recent data showed that the presence of β-sitosterol reduces malondialdehyde overproduction during liver injury [24].

**Figure 2:**
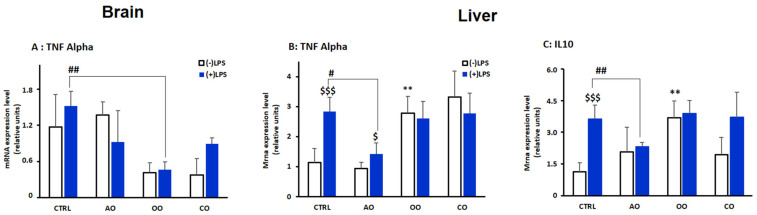
Effect of argan oil, olive oil, or colza oil treatment on gene expression of the proinflammatory marker *Tnf-α* (**A**,**B**) in the brain and liver, respectively, and on the anti-inflammatory marker *Il-10* in the liver (**C**). Male C57BL/6 mice received for 28 days a standard diet (control (CTRL)), a diet enriched with 6% (*w*/*w*) AO, OO, or CO, and intravenous injection of LPS (100 µg) four hours antemortem. First, total RNA was isolated from mice brains or livers, and then the expression level of genes of interest was quantified by real-time RT-qPCR. All values are means ± SD (*n* = 3), Statistical significance of higher mean signal strength (** *p* ≤ 0.01) compared to control, (## *p* ≤ 0.01. # *p* ≤ 0.05) Compared to LPS and ($$$ *p* ≤ 0.001. $ *p* ≤ 0.05) compared to the different treatments with or without LPS administration.

Transcriptional levels of the proinflammatory marker tumor necrosis factor-α (*Tnf-α*) were determined in the brain and liver (Figure 2A,B). Intriguingly, LPS treatment did not significantly affect brain *Tnf-α* mRNA levels (Figure 2A). Of note, the basal expression level of *Tnf-α* was reduced in OO- and CO-treated mice (Figure 2A). By contrast, in the liver, LPS significantly increased (*p* < 0.01) TNFα levels in control mice. This LPS transcriptional effect was prevented by AO pretreatment (*p* < 0.05) (Figure 2B). Pretreatments with either OO or CO increased liver *Tnf-α* mRNA content. In these conditions, the LPS-dependent increased expression of *Tnf-α* was not found (Figure 2B).

Anti-inflammatory interleukin-10 (IL-10) mRNA levels in the brain but not the liver were below the detectable threshold. In the liver, LPS treatment also increased the mRNA expression of *Il-10* (*p* < 0.01). Only OO treatment significantly induced *Il-10* mRNA level (*p* < 0.01), while AO or CO treatment did not show significant changes (Figure 2C). Both OO-LPS and CO-LPS groups showed similar levels compared to the LPS-treated group. In comparison, AO-LPS conveyed a downregulation of *Il-10* mRNA expression compared to the LPS-treated group (*p* < 0.01) (Figure 2C).

### 2.2. Brain and Liver Gene Expression of Peroxisomal Protein-Encoding Genes

**Figure 3:**
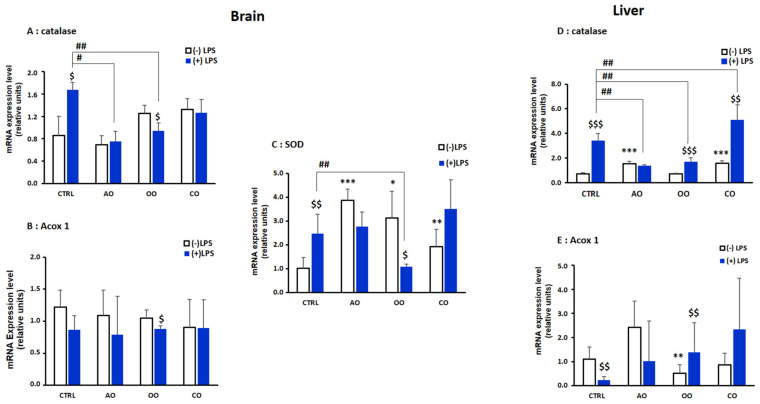
Effect of argan oil, olive oil, or colza oil treatment on gene expression of *Cat* (**A**,**D**) and *Acox1* (**B**,**E**) in brain and liver, respectively, and *Sod* in the brain (**C**). Male C57BL/6 mice received for 28 days a standard diet (control (CTRL)), a diet enriched with 6% (*w*/*w*) AO, OO, or CO, and intravenous injection of LPS (100 µg) four hours antemortem. First, total RNA was isolated from mice brains and livers, and then the expression level of genes of interest was quantified by real-time RT-qPCR. All values are means ± SD (*n* = 3), Statistical significance of higher mean signal strength (*** *p* ≤ 0.001. ** *p* ≤ 0.01. * *p* ≤ 0.05) compared to control, (## *p* ≤ 0.01. # *p* ≤ 0.05) Compared to LPS and ($$$ *p* ≤ 0.001. $$ *p* ≤ 0.01. $ *p* ≤ 0.05) compared to the different treatments with or without LPS administration.

Next, we investigated by RT-qPCR the effect of LPS and oil treatments on the expression of three peroxisomal genes encoding for CAT, SOD1, and ACOX1. In both the brain and liver, catalase mRNA expression was significantly induced by LPS (Figure 3A,D). However, only in the livers of OO and CO treatment groups, *Cat* mRNA was induced (*p* < 0.001) (Figure 3A,D). Pretreatment with AO or OO counteracted the LPS-induced catalase induction in the brain as well as in the livers of AO-LPS treated group and the OO-LPS group, respectively (*p* < 0.01) (Figure 3A,D). In the brain, *Sod1* mRNA expression (Figure 3C) was upregulated in all the oil treatment (AO and OO groups (*p* < 0.01), CO (*p* < 0.05)) (Figure 3C) and were induced further by LPS challenge. The OO-LPS treated group exhibited a significant decrease (*p* < 0.01) in *Sod1* mRNA expression compared with the LPS-treated group (Figure 3C).

Brain *Acox1* mRNA expression did not show significant changes after LPS administration or oils treatments alone (Figure 3B). In response to LPS injection, hepatic *Acox1* mRNA level was significantly reduced (*p* < 0.001) in the LPS vs. control group. *Acox1* mRNA expression was significantly downregulated after OO treatment, while there was a non-significant increase after AO treatment, and after CO treatment there was no change (Figure 3E). *Acox1* mRNA expression did not significantly increase in oil-pretreated animals injected with LPS (AO-LPS, OO-LPS and CO-LPS groups) vs. LPS group (Figure 3E).

### 2.3. Brain and Liver Expressions of Peroxisomal Proteins

Levels of peroxisomal proteins involved in the fatty acid β-oxidation (ABCD1, ABCD2, and ACOX1) or H_2_O_2_-degrading enzyme (CAT) were assessed by immunoblotting. ACOX1, a 72 kDa polypeptide, is structurally imported into peroxisomes and partially processed into 51 and 21 kDa protein products. ACOX1 exists as a dimer, composed of only 72 kDa polypeptides or a combination of 72, 51, and 21 kDa polypeptides [25]. Therefore, an increased signal for 51 kDa peptide could reflect increased processing of the 72 kDa parent protein.

#### 2.3.1. Brain

**Figure 4:**
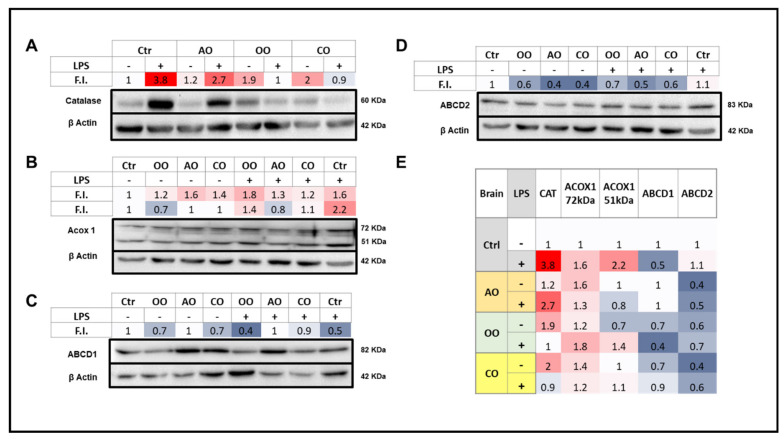
Effect of argan oil, olive oil or colza oil treatment on brain expressions of peroxisomal proteins, CAT (**A**), ACOX1 (**B**), ABCD1 (**C**), ABCD2 (**D**) and the heatmap for all protein expression (**E**). Male C57BL/6 mice received for 28 days a standard diet (control (CTRL)), a diet enriched with 6% (*w*/*w*) AO, OO, or CO, and intravenous injection of LPS (100 µg) four hours antemortem. Brain homogenates were separated in PAGE-SDS electrophoresis and subjected to immunoblotting as described in Section 4. Band intensities were analyzed by densitometry and standardized to β-actine expression level. Tables represent the standardized densitometric analysis obtained after signal intensity quantification of different proteins.

LPS treatment increased brain expression of catalase and both ACOX1 72 and 51 kDa peptides, reduced the expression of ABCD1, and did not affect ABCD2 protein levels (Figure 4). Brain catalase protein expression was increased by OO or CO treatment and to a lesser extent by AO treatment. However, AO was the only oil that did not abolish the LPS-induced rise in catalase levels, and instead maintained the bulk of it (Figure 4). AO, CO, and to a lesser extent OO treatments increased the ACOX1 72 kDa level, while none of the three oils was able to increase the levels of ACOX1 51 kDa polypeptide. Both ACOX1 72 and 51 kDa peptide levels were induced by LPS administration; the highest induction of the 51 vs. 72 kDa peptide suggested a rise in the putative ACOX1 processing rates. LPS-AO and LPS-CO pretreated mice showed a lesser increase in brain ACOX1 72 kDa, in contrast to OO-LPS mice that exhibited net inductions of both ACOX1 72 and 51 kDa polypeptides (Figure 4).

For ABCD1 protein levels in the brain, AO treatment alone and pretreatment in the presence of LPS could maintain control baseline levels, whereas all other experimental conditions led to a decrease in brain ABCD1 levels (Figure 4). For ABCD2, each treatment with one of the three oils decreased the protein levels; these decreases, as well as control levels, were not affected by LPS (Figure 4).

#### 2.3.2. Liver

**Figure 5:**
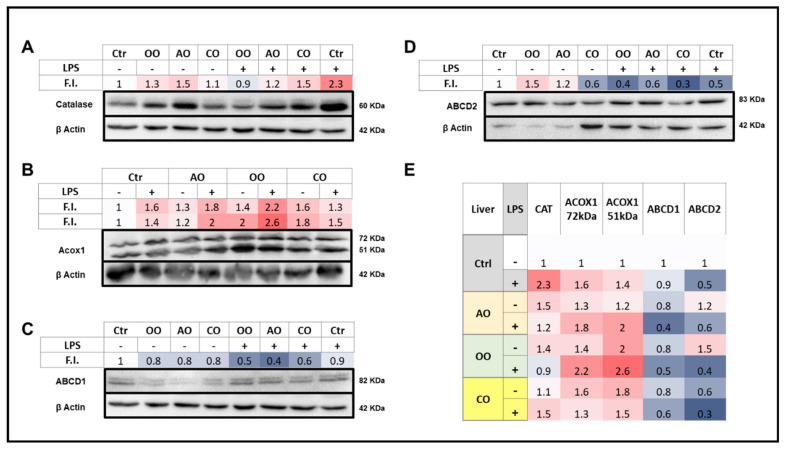
Effect of argan oil, olive oil or colza oil treatment on liver expressions of peroxisomal proteins, CAT (**A**), ACOX1 (**B**), ABCD1 (**C**), ABCD2 (**D**) and the heatmap for all protein expression (**E**). Male C57BL/6 mice received for 28 days a standard diet (control (CTRL)), a diet enriched with 6% (*w*/*w*) AO, OO, or CO, and intravenous injection of LPS (100 µg) four hours antemortem. Liver homogenates were prepared as described in Section 4. Band intensities were analyzed by densitometry and standardized to β-actin expression level. Tables represent the standardized densitometric analysis obtained after signal intensity quantification of different proteins.

LPS treatment induced both CAT and ACOX1 protein levels in the liver to a lesser extent than in the brain. On the other hand, AO or OO oil treatment increased the hepatic expression of catalase, while CO oil did not (Figure 5). ACOX1 72 kDa was augmented by each oil treatment alone and LPS. The 51 kDa protein levels and hence the processing of the 72 kDa was substantially induced by OO or CO treatments better than AO treatment. ACOX1 51 kDa processing was increased by LPS which exacerbated the increase by AO or OO but not CO pretreatment (Figure 5).

Interestingly, ABCD1 protein expression in the liver was largely repressed with the oil treatments, and this effect was accentuated in pretreated mice in the presence of LPS, with little or no effect towards control baseline values. For ABCD2, results were more contrasted, showing a hepatic induction of its expression by AO or OO treatment and repression by CO and by LPS administration with or without oil pretreatment (Figure 5).

### 2.4. Brain and Liver Peroxisomal Antioxidant Enzymes Activities

**Figure 6:**
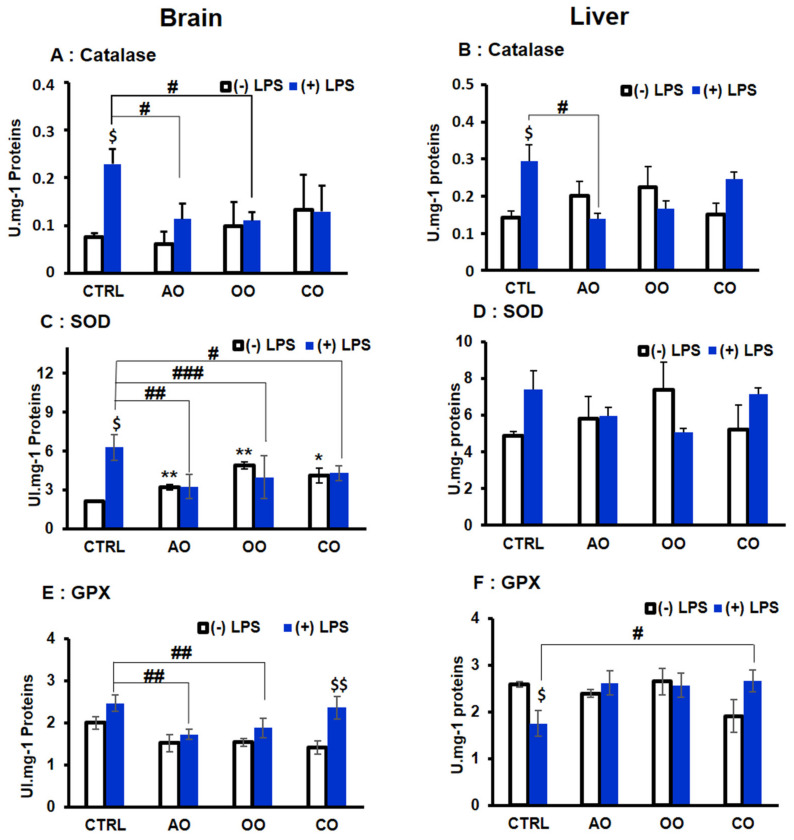
Effect of argan oil, olive oil or colza oil treatment on the antioxidant enzymes activities of CAT (**A**,**B**), SOD (**C**,**D**), and GPX (**E**,**F**) in brain and liver, respectively. C57BL/6 mice received for 28 days a standard diet (control (CTRL)), a diet enriched with 6% (*w*/*w*) AO, OO, or CO, and intravenous injection of LPS (100 µg) four hours antemortem. Brain and liver homogenates were prepared as described in Section 4. Results are expressed in (UI.mg^−1^ = µmol of substrate transformed/minute/mg of proteins). All values are means ± SD (*n* = 3), statistical significance of higher mean signal strength (** *p* ≤ 0.01. * *p* ≤ 0.05) compared to control, (### *p* ≤ 0.001, ## *p* ≤ 0.01, # *p* ≤ 0.05), compared to LPS and $$ *p* ≤ 0.01, $ *p* ≤ 0.05) compared to treatment with or without LPS administration.

The catalytic activities of three antioxidant enzymes CAT, SOD, and GPx were measured in both the brain and liver from the different groups of mice. In the brain, LPS significantly increased the activity of CAT and SOD by 305% and 295%, respectively (*p* < 0.05) (Figure 6A,B). In the liver, LPS significantly doubled catalase activity (205%; *p* < 0.05), while its 1.5-fold increase effect on SOD activity remained statistically non-significant (*p* < 0.07) (Figure 6D). In both tissues, the treatment with AO, OO, or CO alone showed no significant effect on the catalase activity compared to the control group (Figure 6A,B). Interestingly, each oil significantly increased in the brain, but not in the liver, the SOD activity (151% and 229% *p* < 0.01) for AO and OO treatments, respectively, and CO (192% *p* < 0.05) (Figure 6B,D). However, LPS-dependent increase in catalase activity was significantly entirely abolished by pretreatment with AO (LPS-AO group) and OO (LPS-OO group) but not CO pretreatment (Figure 6A,B). In contrast, SOD activity decreased in the brain by each oil pretreatment (68%; *p* < 0.05) compared to the LPS-treated groups (Figure 6C,D).

GPX activity was slightly but not significantly increased in brain homogenates by 123% in the LPS-treated group (Figure 6E). On the contrary, in the liver (Figure 6F), GPX activity was decreased (68%; *p* < 0.05) by the LPS treatment compared to the control group. However, none of each oil (AO, OO, or CO) pretreatment has shown a significant effect on GPX activity. However, in both tissues, these effects were prevented by oil pretreatment. In the brain, LPS-dependent increase in GPX activity (LPS group) was significantly attenuated by AO (70%, *p* < 0.01) and OO (76%; *p* < 0.01) (AO-LPS and OO-LPS groups, respectively) (Figure 6E). In the liver, only CO pretreatment (CO-LPS group) induced a significant increase in GPX activity (151%; *p* < 0.05) compared to the LPS-treated group (Figure 6F).

## 3. Discussion

In previous studies, we have collected compelling evidence of the protective mechanisms of AO against LPS-induced liver injury [10,18]. However, the potential protective effect of AO against LPS-induced brain oxidative stress and peroxisome dysfunction has not been investigated so far. In the present in vivo study, the protective properties of AO (compared to those of OO and CO) have been explored in the scope of a short-term LPS challenge with a focus on antioxidant mechanisms (GSH, antioxidant enzymes, ACOX1).

The non-enzymatic GSH antioxidant defense level was evaluated as an essential component of the intracellular redox balance and cellular biological functions [26]. Four hours after intravenous LPS injection, hepatic GSH levels increase rapidly, which seems to be maintained for four hours and up to sixteen hours post LPS injection [10]. It is well known that during endotoxemia, the liver serves as the primary source of plasma GSH, and this may explain the hepatic increase in GSH level after LPS injection [27]. However, under oxidative stress, this LPS-dependent GSH induction could depend on hepatic macrophage-derived Kupffer cells and not from endothelial cells [4]. Accordingly, previous studies suggested that LPS triggers glutathione synthesis de novo in Kupffer cells [28,29]. By contrast, brain GSH level was downregulated by LPS, as also reported [30] in mice four hours post-LPS injection. The decrease in brain GSH levels could be related to declining microglial cells. These brain phagocytes resident cells regulate brain homeostasis and control neuroinflammation [31]. Thus, LPS-treated mice displayed activated and reactive microglia, particularly in the substantia nigra, leading to neuronal damage [32].

Moreover, activating both enriched primary microglial cultures and the N11 microglial cell line by LPS/Interferon leads to a 40% decrease in GSH microglial content [33]. Here, we show that only AO (but not OO and CO) pretreatment prevented LPS-dependent GSH imbalance by counteracting both the LPS-induced downregulation in the brain and augmentation in the liver. This phenomenon may be linked to the antioxidant capacity spectrum of AO, which contains, in addition to polyphenols and tocopherols, a higher content of coenzyme Q10 and melatonin [34]. Notably, the latter has been shown to induce the expression of γ-glutamylcysteine synthetase, the rate-limiting enzyme of GSH synthesis [35].

On the other hand, lipid peroxidation is defined as the process under which oxidative stress-generated free radicals peroxidize polyunsaturated fatty acids esterified in cell membrane phospholipids [30]. Malonaldehyde, a well-known biomarker for lipid peroxidation [36,37], revealed that short-term four hours LPS injection via the tail vein led to a significant increase in lipid peroxidation in both brain and liver tissues. Reportedly, data show that LPS injected intraperitoneally led to a significant increase in cerebral and hepatic MDA production after 4 h in mice [30] and after 2 h in rats [38], respectively. These increased lipid peroxidation levels are maintained 12 h after intraperitoneal LPS administration [38,39]. We found that the pretreatment with oils prevents the effect of LPS on lipid peroxidation. However, AO showed a more pronounced decrease in MDA production in both brain and liver tissues. As mentioned above, it is noteworthy that AO harbors one of the highest antioxidant capacities among edible oils, including melatonin, coenzyme Q_10_, tocopherols, and ferulic acid [11,37,38,39,40,41,42,43,44]. The antioxidant nature of AO effects may result from its unique composition in antioxidants and unsaturated fatty acids, which contribute to cell membranes stabilization and repair of brain and liver tissue damage caused by LPS [40]. As reported for several edible oils, the unsaturated fatty acids display also antioxidant properties [41]. Earlier work of Khalouki et al. [12] reported the antioxidant profile of argan oil, showing that AO phenolic compounds exhibit an antioxidant capacity superior to the soluble form of vitamin E, Trolox [12]. Total content of antioxidants (range between 456 and 1409 mg/kg) was reported by López et al. [34], including co-enzyme Q_10_, melatonin, polyphenols and tocopherols. Furthermore, in our previous study, we have measured antioxidant properties of argan and extra virgin olive oils by Kit Radicaux Libres (KRL) and ferric reducing antioxidant power (FRAP) tests [42].

Regarding the inflammatory status after LPS injection, *Tnf-α* gene expression increased enormously in the liver and only slightly decreased in the brain. Although brain *Tnf-α* expression moderately affects the oils, this downregulation effect is statistically significant for AO. LPS is a potent activator of *Tnf-α* mRNA and protein production, and its accumulation in murine macrophages [43]. Interestingly, AO-pretreated mice exhibit a significantly limited induction of *Tnf-α* mRNA when compared to OO- or CO-pretreated mice. Correspondingly, Dopp et al. (2002) and Persson et al. (2006) reported that *Tnf-α*-treated primary microglial cells exhibit an increase in GSH level concomitantly to a significant diminution in ROS levels [44,45]. However, oligodendrocytes exhibit an opposite response to *Tnf-α*. Thus, the mechanisms related to the effect of AO and other oils need more investigation to understand the outcome of different brain cell types.

Findings of the present study also include the marked increase in liver *Il-10* mRNA expression in the LPS-treated group. *Il-10* is known to have both inflammatory and anti-inflammatory actions [46]. Our data are in agreement with the reported results [47]. Therefore, we suggest that the rise in *Il-10* mRNA expression represents an anti-inflammatory response to counteracting the hepatic inflammation induced by LPS injection. Accordingly, the combined AO and LPS treatment showed a significant decrease in hepatic *Il-10* mRNA expression compared with the LPS alone treated group, which may attest to the anti-inflammatory role of AO [10].

A partial or complete defect of the peroxisomal compartment is associated with severe inherited metabolic disorders (e.g., Zellweger syndrome and X-ALD) [6,8,48]. The overall cellular impact of the resulting peroxisomal metabolism dysfunction encompasses the increases in both endoplasmic reticulum and mitochondrial stresses [49,50,51,52]. The present study explored the brain and liver peroxisomal antioxidant and fatty acid β-oxidation during sepsis and their potential restoration by AO. LPS induced both brain *Cat* and *Sod1* mRNA expressions, and a similar result was recorded for *Cat* mRNA in the liver. Imbalanced expression of catalase in mice results in enhanced NF-κB activation and inflammation and excessive injury to different tissues [53].

Furthermore, it is well established that LPS provokes an increase in SOD mRNA expression [54,55,56]. Co-treatment with AO, and to a lesser extent with OO, inhibited the LPS effects in both the brain and the liver to control levels, CO oil failing to normalize the level of *Cat* mRNA expression in the brain and preserving this level in the liver. The effect of AO might be, among others as mentioned above, related to its highest content in PUFAs, which have been reported to induce catalase in hepatic cells [57]. The up-regulation of PPARα by PUFA or palmitoylethanolamide ligands is likely to control peroxisomal catalase and SOD [58,59]. In light of this, and as we previously reported, AO treatment triggers the expression of PPARα, its target genes, and its coactivator PGC-1α in mouse liver [18].

Regarding the enzymatic activity level, our results indicate that LPS injection triggered the induction of brain peroxisomal enzymatic antioxidants activities such as CAT, SOD, and GPX. However, a decrease in GPX activity is observed in the liver, and LPS administration may lead to an essential decrease in antioxidant enzyme activities [60,61,62]. As discussed previously, the increase in the peroxisomal antioxidant enzymes activity can be seen as part of a defense mechanism against cellular free radicals [19,63,64]. Interestingly, LPS dysregulation in the brain and liver antioxidant status were prevented when mice were pretreated with AO, OO, and CO. Furthermore, the protective effect was more pronounced, with AO more than OO and CO in preserving brain CAT, SOD, and GPX activities to control values. As mentioned above, the antioxidant capacity and spectrum of AO are attributed to its high content in phenolic compounds and tocopherols (α-, β-, γ- and δ-tocopherols) [16,65]. Several studies have highlighted a rich chemical composition of AO, which, in terms of antioxidant compounds, encompasses sterols, tocopherols, polyphenols, and carotenes, all endowed with strong free radical scavenging properties and robust biological/pathogenesis antioxidant protection [12,15]. Interestingly, in vitro bioavailability of polyphenols and antioxidant properties of AO and OO were evaluated by Seiquer et al. [66], showing an increase in polyphenol content and antioxidant activity during AO digestion. Furthermore, intestinal Caco-2 cells absorb large proportion of phenol compounds and antioxidant capacity [66]. Some AO compounds (i.e., fatty acids, phytosterols, polyphenols, tocopherols) that can cross the blood–brain barrier may be neuroprotective [19]. Accordingly, several dietary phenolic compounds, including curcumin, the yellow pigment in turmeric, and the green tea flavanol have been shown to exert neuroprotection through the activation of the nuclear receptor Nrf2 (Nuclear Factor (Erythroid-Derived 2)-Like 2) and its cytoprotective target genes [67].

Additionally, we have investigated *Acox1* mRNA expression in the liver. *Acox1* encodes the first and rate-limiting enzyme in the peroxisomal β-oxidation pathway. Its deficiency leads to the accumulation of very-long-chain fatty acids (VLCFAs) and a substantial reduction in peroxisome abundance [68]. Our results showed significant repression by LPS of hepatic *Acox1* mRNA level and no significant effect on brain *Acox1* transcript. AO and OO showed no effect against this repression, and only CO was shown to significantly enhance the *Acox1* mRNA expression. These results are in agreement with previous studies [18,69]. However, at the protein level, ACOX1 protein expression was induced in mice brain and liver treated with LPS alone or pretreated with oil alone (i.e., AO, OO, or CO) or in the presence of both. An intriguing discrepancy was observed between transcriptional and translational regulations due to differences in mRNA and protein decay rates, knowing that aberrant translation can accelerate mRNA decay [63]. On the other hand, protein translation is under the control of the PERK/eIF2α-P/ATF4 signaling, which inhibits the decline of protein synthesis during endoplasmic reticulum stress provoked by LPS [64]. Additionally, the peroxisomal fatty acid β-oxidation pathway induction by PPARα-dependent PUFA activation has been primarily documented [6], and this may explain the enhanced ACOX1 protein expression by AO, which encompasses the highest PUFA content [11,70]. Accordingly, we have previously shown that AO regulates hepatic fatty acid oxidation pathways through the activation of the nuclear receptors -PPARα, ERRα- and their coactivator, PGC-1α [18].

The activity of ACOX1 is dependent on the supply of its substrates (i.e., fatty acids and fatty acyl-CoAs), including transport into the peroxisomal compartment. ABCD1 and ABCD2, located in the peroxisomal membrane, are involved in transporting VLCFA-CoAs into the peroxisome prior to their β-oxidation [71]. Our results showed that LPS treatment had a moderate decrease effect on ABCD1 protein expression in the brain, and this effect was relatively counteracted by AO and CO supplemented diets. On the other hand, LPS did not affect ABCD2 expression in the brain. Furthermore, LPS treatment did not affect ABCD1 protein expression in the liver but decreased ABCD2 protein level. In addition, we have previously reported that knocking of Abcd1 or Acox1 mRNA expression in oligodendrocytes resulted in an increased overproduction of ROS by VLCFA [72].

Interestingly, AO pretreatment rescued the brain protein expression of ABCD1 and was also shown to induce *Abcd1* mRNA level [42]. However, ABCD1 dysfunction results in defective transport of VLCFAs into peroxisomes and hence reduced adequate availability for their β-oxidation. Consequently, this may lead to increased phospholipids and cholesterol esters containing VLCFAs, which cause severe neurodegenerative disease in the central nervous system [73].

Collectively, our data showed that the AO protective effects against short-term LPS-induced brain and liver oxidative stress through the preservation of the peroxisomal antioxidant and fatty acid β-oxidation functions. Regarding our previous work on liver injury induced after 16 h LPS post-injection [10], here we showed that the hepato-protective action of AO is effective at short-term (i.e., 4 h), by normalizing hepatic peroxisomal antioxidant and β-oxidative capacities. For the first time, we demonstrated that AO also has a neuroprotective effect during sepsis. In the future, a combination of lipidomic and transcriptomic analysis would clarify the metabolic circuits involved in the AO hepato- and neuro-protective effects against LPS. Additionally, it would be interesting to explore the potential effects of individual AO constituents, such as polyphenols. This may bring new arguments to the potential beneficial role of AO in the management of sepsis and as an alternative therapy to synthetic chemical compounds that may have several adverse effects.

## 4. Material and Methods

### 4.1. Chemicals and Reagents

RNeasy Mini kit (Qiagen, Courtaboeuf, France), iScript cDNA Synthesis Kit (Bio-Rad, Marnes-la-Coquette, France) MESA GREEN qPCR MasterMix Plus (Thermo Fischer Scientific, Illkirch, France), Applied Biosystem Step One QPCR machine (Thermo Fischer Scientific, Illkirch, France), Potter Elvehjem homogenizer (Dominique Dutscher, Issy-les-Moulineaux, France), Anti-ABCD1 and anti-ACOX1 (BioPeroxIL laboratory, Dijon, France), Anti-ABCD2 (ab 102948, Abcam, Cambridge, UK), anti-catalase (AF3398, R&D Systems, Minneapolis, MN, USA). Other chemicals were purchased from Sigma-Aldrich (Saint-Quentin-Fallavier, France).

### 4.2. Mice Treatments

C57BL/6 J male mice (12–16 weeks old, 28–32 g body weight) were purchased from Pasteur medical Laboratory in Casablanca, Morocco. Mice were used under the recommendations of the Organization for Economic Co-Operation and Development (OECD), ‘Test no. 407: repeated dose 28-day oral toxicity study in rodents,’ 1995). All animal experiments were approved by the ethic committee of the faculty of medicine of Hassan II University and carried out according to ethical rules of the University of Hassan I, according to the National Institutes of Health guide for the care and use of Laboratory Animals (NIH publication No. 85-23, revised 1985) for the care and the use of laboratory animals. All mice were housed in a pathogen-free environment under a light-dark (12 h–12 h) cycles, relative humidity (45–65%), at a temperature of 22 ± 2 °C, and fed with standard diet and water ad libitum. After 10 days of acclimatization, the mice were randomly divided to eight groups (5 mice/group), each group receiving for 28 days a standard diet added or not with a vegetal oil: 2 control groups fed with a standard diet; 2 argan oil groups fed with a standard diet supplemented with 6% (*w*/*w*) AO; 2 olive oil groups fed with a standard diet supplemented with 6% (*w*/*w*) OO and 2 colza oil groups who received a standard diet supplemented with 6% (*w*/*w*) CO. Each oil was solubilized in acetone (1:3 *v*/*v*), add to diet pellets and evaporated overnight. Four hours before euthanasia and during the fed state, one group from each group (control (+LPS), AO (AO + LPS); OO (OO + LPS) and CO (CO + LPS)), received an injection (5 mg/kg) via tail vein of 100 µg of *Escherichia coli* 0111:B4 LPS (Sigma, Saint-Quentin-Fallavier, France), solubilized in sterile phosphate-buffered saline (PBS) or an equal volume of PBS alone [69]. After euthanasia, brain and liver tissues were immediately frozen in an ethanol-dry ice bath and stored at −80 °C.

### 4.3. Origin, Extraction and Composition of Oils

Argan oil (AO) is a virgin edible oil which was extracted by mechanical cold-pressing roasted kernel of argan (*Argania spinosa* (L.) Skeels; Sapotaceae), an endemic tree to Morocco. Argan oil and olive oil (OO) (*Olea europaea* L. cv. Moroccan picholine) were obtained from Aklim region, latitude: 34°55′45″ North; longitude: 2°26′7″ west, Berkane, Morocco. Colza oil (CO) (*Brassica napus* subsp. Napus) was obtained from a commercial supermarket.

Fatty acid methyl esters were prepared as described before [12,74]. Briefly, 0.3 g of oil sample mixed in a heptane and methanolic KOH (2 M) solution, was analyzed by gas chromatography (GC; HP 6890, Agilent Technologies, Santa Clara, CA, USA) supplied with a flame ionization detector and a capillary column (Carbowax 20 M, 30 m × 0.32 mm, 0.25 μm thickness, Agilent Technologies, Santa Clara, CA, USA). The nitrogen flow rate was at 2.5 mL/min and the temperature from 140 to 240 °C at 10 °C/min. Identification of fatty acids was based on their retention times and those of standards.

Phytosterols analysis were performed as reported by El Kharrassi et al. [74]. Briefly, oil sample (2.5 g) was diluted in 30 mL ethanolic KOH (2 M) solution. This mixture was maintained during 30 min at light boiling, then combined with distilled water (25 mL) and petroleum ether (75 mL). The obtained organic phase was washed with distilled water, evaporated in a rotary evaporator, and then supplemented with 1 mL hexane and spotted on a thin-layer chromatography (TLC, Biomnis, France). The plate was developed in a solvent solution of hexane/diethyl ether (66:34, *v*/*v*). Then, we dissolved phytosterol extracts in 10 mL chloroform. The dried extract was silylated with 500 μL of a pyridine/hexamethyldisilazane/trimethylchlorosilane (9:3:1, *v*/*v*/*v*) mixture, evaporated under nitrogen flow. The analysis was performed in a HP-5 integrated silica capillary column (15 m × 0.25 mm, 0.25 μm thickness) in a gas chromatograph (GC; HP 6890, Agilent Technologies, Santa Clara, CA, USA).

Tocopherols were identified as reported by El Kharrasi et al. [74] using a high-performance liquid chromatography (HPLC, HP 1050, Hewlett–Packard, Avondale, PA, USA) equipped with an HP 1047A differential refractometer detector, and an octadecylsilane silica (C18) column. Elution of tocopherols was achieved by an isooctane/isopropanol (99:1, *v*/*v*) solvent.

### 4.4. Tissue Homogenates Preparation for Enzymatic Activity Assays

Homogenates were prepared by grinding animal’s livers and brains in 10% (*w*/*v*) for the livers and 4% (*w*/*v*) for the brain potassium phosphate buffer 0.05 M, pH 7.4, respectively. Tissues were homogenized using potter Elvehjem homogenizer (Dominique Deutscher, Issy-les-Moulineaux, France). Homogenates were centrifuged for 10 min at 3000× *g* at 4 °C and supernatants considered as extracts were stored at −20 °C. Protein content was measured using bovine serum albumin as a standard, according to the method described by Lowry et al. [75].

### 4.5. Measurement of Enzymatic Activities

#### 4.5.1. Determination of Catalase Activity

Catalase activity was determined as described before [76]. The assay mixture containing 10 µL of the extracts and 30 µL of H_2_O_2_ (7.3 mM solution) was incubated on ice for 5 min. The reaction was then quenched by adding 20 µL of sulfuric acid H_2_SO_4_, 6 N. The amount of H_2_O_2_ remaining in the reaction mixture after 5 min of catalase action was determined by titration with 140 µL of 2 mM potassium permanganate (KMnO_4_). The rate of decomposition of H_2_O_2_ was measured by spectrophotometry at 480 nm. The specific catalase activity is expressed as µmole/min/mg of protein.

#### 4.5.2. Determination of Superoxide Dismutase Activity

Superoxide dismutase activity was assessed by the method described before [77]. This method involves the inhibition of tetrazolium nitroblue reduction (NBT) using riboflavin as a superoxide generator. The homogenate (20 µL) was mixed in a 50 mM phosphate buffer containing 0.025% Triton X-100, 75 mM nitro blue tetrazolium chloride NBT, 0.1 mM EDTA pH 8 and 12 mM L-methionine. The reaction is initiated by adding 2 µM riboflavin at room temperature, and the absorbance is read at 560 nm.

#### 4.5.3. Determination of Glutathione Peroxidase Activity

Glutathione peroxidase activity was measured following the method of Flohé and Günzler [78]. Briefly, homogenate (300 µL) was incubated for 15 min at 37 °C in a reaction mixture containing 300 µL of potassium phosphate buffer (0.1 M, pH 7.0), 200 µL GSH (reduced glutathione, 2 mM), 100 µL H_2_O_2_ (1 mM) and 100 µL sodium azide (1 mM). Then, 0.5 mL of TCA (5%) was added to stop the reaction. After centrifugation for 5 min at 1500× *g*, 100 µL of the supernatant was collected and added to 200 µL of phosphate buffer (50 mM, pH 7.0) and 0.7 mL of (5,5-dithiobis (2-nitrobenzoic acid; DTNB) buffer (0.4 mg/mL). The absorbance was measured at 420 nm.

### 4.6. Determination of Reduced Glutathione Level

The level of reduced glutathione was evaluated by the method of Ellman [79]. Brains or livers homogenates (400 µL) were mixed with Trichloroacetic acid (5%) and centrifuged at 12,000× *g* for 10 min. The supernatant (50 µL) was diluted in 850 µL of phosphate buffer (50 mM, pH 8) and 100 µL of DTNB (6 mM) was added. The absorbance was read at 412 nm within 5 min against a blank containing the same reagents. The GSH concentrations were calculated by using a standard glutathione curve.

### 4.7. MDA Level Evaluation

Lipid peroxidation was measured via the thiobarbituric acid-colored reaction as described before [80]. The reaction mixture containing 0.5 mL of the homogenate, 0.5 mL of trichloroacetic acid (TCA, 20%) and 1 mL of thiobarbituric acid (TBA, 0.67%) was heated at 100 °C for 15 min. After cooling, MDA was extracted with *n*-butanol; centrifugation at 3000× *g* for 15 min was carried out, and the absorbance of the organic layer was measured at 532 nm.

### 4.8. Quantification of Gene Expression by RT-qPCR

Total RNA from brain and livers tissues was isolated using the RNeasy Mini kit (Qiagen, Courtaboeuf, France) following the manufacturer’s instructions. Total mRNA concentration was measured by spectrophotometry at 260 nm using a TrayCell (Hellma, Paris, France). The purity of nucleic acids was checked by measuring the ratio of the absorbance 260 nm/280 nm, between 1.8 and 2.2. Next, one µg of mRNA was reverse transcribed to generate cDNA using iScript cDNA Synthesis Kit (Bio-Rad). The cDNAs were then used for quantitative PCR analysis of specific genes (Thermo Fischer Scientific). All PCR reactions were performed in triplicate, using the MESA GREEN qPCR MasterMix Plus (Thermo Fischer Scientific), on an Applied Biosystem Step One QPCR machine (Life Science Technologies). The primers sequences are detailed in Table 2. Thermal cycling conditions were as follows: activation of DNA polymerase at 95 °C for 10 min, followed by 40 cycles of amplification at 95 °C for 15 s, 60 °C for 30 s, and 72 °C for 30 s. At the end of the reaction, a melting curve analysis was calculated to test the absence of non-specific products. For each transcript, the amplification efficiency was determined by the slope of the standard curve generated from two-fold serial dilutions of cDNA. Gene expression was quantified using cycle threshold (Ct) values and normalized by the 36B4 reference gene. Relative expression of genes was determined according to 2^−ΔΔCt^ method.

### 4.9. Immunoblotting

Mice brains and livers were lysed in 4% (*w*/*v*) or 10% (*w*/*v*) RIPA buffer (50 mM Tris-HCl, pH8.0, 150 mM NaCl, 1% NP-40, 0,1% SDS, 0.5% sodium deoxycholate), respectively, using a potter Elvehjem homogenizer (Dominique Deutscher, Issy-les-Moulineaux, France). The lysates were centrifuged at 10,000× *g* for 10 min at 4 °C, and the supernatants were collected. Their protein content was determined using the Bicinchoninic Acid Solution (Thermo Fisher Scientific). Fifty µg of proteins were diluted (*v*/*v*) in the loading buffer (125 mM Tris-HCl, pH 6.8, 4% SDS, 20% glycerol, 14% mercaptoethanol, and 0.003% Bromophenol blue) and heated at 100 °C for 5 min, then separated on a 10% SDS-PAGE, and transferred into PVDF membrane. After blocking non-specific binding sites with 5% nonfat milk in TBST (10 mM Tris-HCl, 150 mM NaCl, 0.1% Tween 20, pH 8) for 1 h at room temperature, the membrane was incubated overnight at 4 °C with the primary antibody diluted in 1% milk TBST (anti-ABCD1,“serum 029” from BioPeroxIL laboratory, [81] dilution 1/2000; anti-ABCD2, ab 102948, ab 102948 from Abcam, dilution 1/1000; anti-catalase, AF3398 from R&D Systems, dilution 1/400; anti-b-actin, A2228 from Sigma-Aldrich, dilution 1/10,000). After three washes for 10 min in PBST, the membranes were incubated for 1 h at room temperature with a secondary appropriate horseradish peroxidase-conjugated antibody diluted in 1% milk TBST (dilution 1/5000). The membranes were then washed three times in TPBS for 10 min. and the immunoreactivity was revealed by enhanced chemiluminescence using the Supersignal West Femto Maximum Sensitivity Substrate (ThermoFisher Scientific) and a Chemidoc XRS+ device (Bio-Rad). Images processing and quantification was performed using the Image Lab software (Bio-Rad).

### 4.10. Statistics

The data is presented as mean values ± SE. Excel (Microsoft, WA, USA) was used for statical Analysis by *t*-test. The differences between the groups analyzed. Statistical significance was defined as a *p* value of less than 0.05.

## Figures and Tables

**Table 1:** Composition of oils.

Fatty Acids (g/100 g Oil)	Argan Oil	Olive Oil	Colza Oil
Myristic acid C14:0	0.1	-	-
Palmitic acid C16:0	13.4	9.09	4.5
Palmitoleic acid C16:1 n-7	0.1	0.73	0.3
Stearic acid C18:0	6.5	2.26	1.6
Oleic acid C18:1n-9	46.4	76.35	63
Linoleic acid C18:2n-6	32.2	9.95	19
Linolenic acid C18:3 n-3	0.1	0.86	9
Arachidic acid C20:0	0.4	0.31	0.5
Eicosenoic acid C20:1	0.4	0.35	1.3
**Phytosterol** (mg/100 g oil)			
Cholesterol	-	0.06	0.3
Brassicasterol	-	-	10.1
Campesterol	0.2	3.21	34.4
Campestanol	-	0.04	-
Stigmasterol	-	1.59	0.2
b-Sitosterol	-	84.77	44.5
Δ-5-Avenasterol	-	5.23	3.1
Δ- 7-Stigmasterol	-	0.24	0.1
Δ-7-Avenasterol	4.2	0.336	0.1
Spinasterol	35.3	-	-
Schottenol	43.8	-	-
**Tocopherol** (mg/Kg oil)			
α-Tocopherol	8.3	87.92	24.03
γ-Tocopherol	88.8	4.62	69.01
δ-Tocopherol	1.2	7.46	6.96

**Table 2:** Sequences of the primers used for PCR.

Gene Name	Primer Sequences
*Il10-F* *Il10-R*	5′ GCTGGACAACATACTGCTAACC 3′
	5′ CCCAAGTAACCCTTAAAGTCCTG 3′
*Acox1-F* *Acox1-R*	5′ TCGAAGCCAGCGTTACGAG3′ 5′ GGTCTGCGATGCCAAATTCC3′
*Tnf α-F* *Tnf α-R*	5′ GACGTGGAAGTGGCAGAAGAG3′ 5′ TGCCACAAGCAGGAATGAGA3′
*Catalase-F* *Catalase-R*	5′ AGCGACCAGATGAAGCAGTG3′ 5′TCCGCTCTCTGTCAAAGTGTG3′
*Sod1-F* *Sod1-R*	5′AACCAGTTGTGTTGTCAGGAC3′ 5′CCACCATGTTTCTTAGAGTGAGG3′
*36b4-F* *36b4-R*	5′CGACCTGGAAGTCCAACTAC3′ 5′ATCTGCTGCATCTGCTTG3′

## Data Availability

Data is contained within the article.
